# Information flow in finite flocks

**DOI:** 10.1038/s41598-020-59080-6

**Published:** 2020-03-02

**Authors:** J. Brown, T. Bossomaier, L. Barnett

**Affiliations:** 1 School of Computing & Mathematics, Charles Sturt University, Bathurst, NSW Australia; 2 Centre for Research in Complex Systems, Charles Sturt University, Bathurst, NSW Australia; 3 Sackler Centre for Consciousness Science, Department of Informatics, University of Sussex, Brighton, UK

**Keywords:** Information theory and computation, Complex networks

## Abstract

We explore information flow in finite active matter flocks by simulating the canonical Vicsek model and estimating the flow of information as a function of noise (the variability in the extent to which each animal aligns with its neighbours). We show that the global transfer entropy for finite flocks not only fails to peak near the phase transition, as demonstrated for the canonical 2D Ising model, but remains constant from the transition throughout the entire ordered regime to very low noise values. This provides a foundation for future study regarding information flow in more complex models and real-world flocking data.

## Introduction

Recent experimental studies of animal flocks, fish^1,2^, birds such as pigeons^3^ and starlings^4^, midges^5^ and sheep^6^ have dramatically increased our understanding of flocking dynamics. A central theoretical issue is how the communication range between flock members leads to global coordination of the flock. We thus measure information flow within flocks using *Transfer Entropy*^7^ and *Global Transfer Entropy*^8^, where the former measures flow between individual agents (stochastic processes) and the latter measures information flow from all agents to a single agent, averaging over all target agents.

Studies of experimental flocks, such as starlings^9,10^, sheep^6^, fish^11^ and midges^5^ lead to proposals^12^ that the flock exists on the boundary between order and disorder—providing the ideal scenario for collective reaction to external stimuli, with enough order to form collective behaviour without overwhelming inertia. A principal finding of this paper is the surprising result that the information flow in the canonical Vicsek flocking model reaches a maximum around the time the flock becomes stable and remains high until very low noise, where noise represents the uncertainty with which an agent aligns with its neighbours.

Real world flocks are *active matter*—systems far from equilibrium, which do not conserve momentum or other dynamical quantities^13^ and there are now realistic models^14^. But to make the computation of continuous global tranfer entropy tractable^15^, we adopt the Vicsek model. With a huge amount of work existing for this model, it could be considered the canonical model for flocking dynamics.

To analyse the long-term limit GTE of the minimalist Standard Vicsek Model (SVM)^16^ of collective motion^17,18^ we developed a closed-form dimensional reduction, obtained by exploiting an approximate isometry in the SVM. This approach has demonstrated *continuously broken ergodicity*^19^ in the Mutual Information^20^, which diverges as noise tends to zero.  While not the object of interest in this study, we note—and discuss briefly in Sections 2 and 4—there are various issues surrounding the precise nature of the phase transition in the Vicsek model. In ongoing work, we are studying the XY spin model, where the spins take continuous values, to which the Vicsek Model converges at zero noise.

While the final closed-form expression for GTE (Eq. (9)) requires an isometry approximation, two technical innovations (Eqs. (6) and (7)) were needed to reduce computational requirements (without isometry): replacing the multi-dimensional vector of interacting particles with a consensus vector; and exploiting the independence of the noise, leading to the surprising result (Eq. (7)) that calculation of global information flow requires *no* measurement of neighbouring particles.

While the claims of flocks at criticality^5,12^ are related to speed fluctuations, of which the Vicsek model has none, this study aims to lay a foundation for how GTE behaves in a continuous, active-matter system. Thus this new behaviour may inform future studies on real-world flocks or other more sophisticated models, noting that for the SVM maximum information flow occurs not just near the phase transition, but throughout the entire low noise regime as well.

## The Standard Vicsek Model

The SVM comprises a set of finite *N* point particles (labelled $i=1,\ldots ,N$) moving on a plane of linear extent *L* with periodic boundary conditions (see Supplementary Material online for full details). Each particle moves with constant speed *v*, and interacts only with neighbouring particles within a fixed radius *r*. Positions ${\overrightarrow{x}}_{i}(t)$ and headings ${\theta }_{i}(t)$ are updated synchronously at discrete time intervals $\Delta t=1$ according to

1 $${\overrightarrow{x}}_{i}(t+\Delta t)={\overrightarrow{x}}_{i}(t)+{\overrightarrow{v}}_{i}(t)\Delta t$$

2 $${\theta }_{i}(t+\Delta t)={\varphi }_{i}(t)+{\omega }_{i}(t)$$

respectively, ${\varphi }_{i}(t)$ is the average heading of all particles within the interaction radius, ${r}_{i}$, of particle *i* (including particle *i* itself), and ${\omega }_{i}(t)$ is white noise uniform on the interval $[\,-\,\eta /2,\eta /2]$ with intensity $\eta \in (0,2\pi ]$. The average heading^16^, which constructs the consensus vector, is ${\varphi }_{i}(t)=\arctan [{\langle \sin (\theta (t))\rangle }_{i,r}/{\langle \cos (\theta (t))\rangle }_{i,r}]$, where ${\langle z\rangle }_{i,r}\equiv \frac{1}{N}\,{\sum }_{j}^{N}\,{z}_{j}{\delta }_{r}(i,j)$ and ${\delta }_{r}(i,j)$ is 1 if neighbour *j* is within the interaction radius, *r*, of particle *i*, and 0 otherwise; note that ${\delta }_{r}(i,i)=1$, so that ${z}_{i}$ is always included in the average. The velocity vector ${\overrightarrow{v}}_{i}(t)$ is constructed from the heading ${\theta }_{i}(t)$ with constant speed *v*. Particle density $\rho =N/{L}^{2}$ is fixed throughout at 0.25.

Considering the SVM as a steady-state *statistical ensemble* containing a finite number of particles^21^ with control parameter $\eta$, we use capitals to indicate quantities sampled from the ensemble; in particular, ${\Theta }_{I}$ denotes the heading of a particle sampled from the ensemble. Specifically, the ensemble is a distribution over the set of all possible realisations of the steady state SVM, *i.e*., any particular realisation has an associated probability (of being sampled), where a realisation here refers to a time series of particle locations and headings, with individual particles identified by an index *i*, and time by an index *t*. ${\Theta }_{I}$ is sampled by first sampling a realisation according to its associated probability, choosing an arbitrary (as we are in the steady state) time stamp, *t*, and then finally sampling a particle index, $I=i$ uniformly from $[1,N]$. Thus the sampled value ${\Theta }_{I}$ is the heading of particle *i* at time *t* in the sampled realisation. Corresponding random variables (discussed below, *i.e*., ${\Theta {\prime} }_{I}$, *etc*.) are sampled in relation to ${\Theta }_{I}$, that is, ${\Theta {\prime} }_{I}$ is the heading of the *same* particle in the *same* realisation as ${\Theta }_{I}$, but at time step $t+1$.

The ensemble comprises multiple realisations (see below), each of which comprises running the model forward in time from random starting conditions for a fixed number of time steps. Each realisation is initialised in the high noise ($\eta =2\pi$) state, with particles distributed uniformly over the simulation plane with uniformly distributed headings. Realisations are simulated for a number of lead-in time steps to allow the system to settle, before all particles and their interactions are captured for *T* time steps and added to the ensemble for noise $\eta$. $\eta$ is then reduced and the process is repeated. It was found the lead-in time steps could be varied with respect to $\eta$, without impact, and so 10^3^ time steps were used for $\eta \ge 3.0$, 5 × 10^4^ time steps for $\eta \le 1.0$ and 2 × 10^4^ time steps otherwise.

The full *order parameter* for the SVM ensemble is the 2D mean particle velocity vector ***M*** with magnitude $M\in [0,1]$ and heading $\Phi \in (0,2\pi ]$. $M=1$ iff all particles are aligned, while in the fully-disordered case ($\eta =2\pi$) we have $M\to 0$ in the large-system limit $N\to \infty$. The ensemble variance

3 $$\chi =[\langle {M}^{2}\rangle -{\langle M\rangle }^{2}]{L}^{2}$$

defines the *susceptibility*; a peak in $\chi$ as a function of $\eta$ is taken to locate an (approximate) phase transition^21^. Technically, this assumes the fluctuation-dissipation theorem^22^, which the SVM does not obey; however, the quantity is widely used in studies of the SVM^21,23,24^.

The phase transition in the original SVM was thought to be second order, but this was disputed^24,25^ and it transpired that seemingly minor details affect the nature of the transition: type of noise statistics^26^; forward versus backward updating (especially at high particle velocities)^27^; boundary conditions associated with density bands or spin waves^28^; and the cone of influence on each particle^29,30^. The issue appeared to be laid to rest, with the SVM transition decided as second order for low velocities, and first order for high velocities^23,31,32^. However recent work has provided a counter-balance, with further evidence for a first-order transition^33^. Consequently, we employ an agnostic, pragmatic approach, utilising the original SVM model (backward updating, angular noise, periodic boundary conditions and low density) over a range of velocities. Observation of the Binder cumulant^34^ for these regimes (not shown) indeed shows a sharp minimum—representative of a first order transition—only at high velocity magnitudes ($v=2.00$), consistent with^23^.

The finite-size SVM exhibits behaviours^20^ akin to “continuously-broken ergodicity”^19^. Over short observation windows the SVM is confined to a comparatively small volume of phase space, thus breaking symmetry and ergodicity. As the window increases, the SVM explores progressively larger volumes of phase space, until ergodicity is restored, albeit requiring very long windows at low noise.

Thus the ensemble statistics are observation time-dependent^20^, giving two regimes—*short-term* and *long-term* statistics. In the short-term regime, we collate statistics (with no ergodic assumptions, and thus only a single realisation per ensemble) over ranges of observation sizes spanning several orders of magnitude, demonstrating the effect of observation time. In the long-term limit, since ergodicity is unbroken, the time average of the GTE will be equal to the ensemble average, and thus statistics can be measured from ensembles constructed of many independent realisations—each with shorter observation windows—rather than the prohibitively long time spans required for solitary realisations to traverse the entire phase space.

## Global Transfer Entropy

The information flow between two continuous (in state; discrete in time) random processes, from $Y$ to $X$, is given by the TE^7^:

4 $${{\mathcal{T}}}_{Y\to X}={\bf{H}}(X{\prime} |X)-{\bf{H}}(X{\prime} |X,Y),$$

where *X*, *Y* are the process histories, and *X*′ is the updated state (*i.e*., a time lag of one). We truncate process histories to just the most recent state as in^8^. For a continuous random variable *X*, ${\bf{H}}(X)\equiv -\,{\int }^{}\,{p}_{X}(x)\,\log \,{p}_{X}(x)dx$ denotes *differential entropy*, which necessitates a continuous estimator^15,35^. GTE extends TE to measure information flow from all particles to a single particle, and here is defined as the ensemble statistic

5 $${{\mathcal{T}}}_{gl}\equiv {{\mathcal{T}}}_{{\boldsymbol{\Theta }}\to {\Theta }_{I}}={\bf{H}}({\Theta {\prime} }_{I}|{\Theta }_{I})-{\bf{H}}({\Theta {\prime} }_{I}|{\boldsymbol{\Theta }})$$

where *I* is uniform on the set of particle indices in the ensemble, ${\Theta {\prime} }_{I}$ is the updated heading at time $t+1$ of the particle indicated by ${\Theta }_{I}$, and ${\boldsymbol{\Theta }}=({\theta }_{1},\ldots ,{\theta }_{N})$ is the vector of all *N* particle headings at time step *t* in the corresponding realisation.

Since the update of a particle’s heading is mediated purely by the consensus heading, ${\Phi }_{I}$, of its neighbours, rather than the whole system, the GTE may be reduced to three dimensions, *i.e*., ${\bf{H}}({\Theta {\prime} }_{I}|\Theta )={\bf{H}}({\Theta {\prime} }_{I}|{\Phi }_{I})$, giving:

6 $${{\mathcal{T}}}_{gl}\equiv {{\mathcal{T}}}_{{\Phi }_{I}\to {\Theta }_{I}}={\bf{H}}({\Theta {\prime} }_{I}|{\Theta }_{I})-{\bf{H}}({\Theta {\prime} }_{I}|{\Phi }_{I}),$$

thus eliminating dimensionality issues surrounding ${\boldsymbol{\Theta }}$ Specifically, using Θ implies N-dimensional coordinates used in the continuous entropy estimator, which subsequently utilises a max-norm distance metric in its estimation to determine bounds for fixed radius searches. Using all particles increases the likelihood of a fixed radius search of *r* = *π*, *i.e.*, all particles.

Noting that, for a single time step as used here, ${\theta {\prime} }_{i}=[{\varphi }_{i}+{\omega }_{i}]$ for any particle *i*—where $[\ldots ]$ denotes modulo 2*π*, confining the result to $(\,-\,\pi ,\pi ]$—and that noise ${\omega }_{i}$ is independent of ${\varphi }_{i}$ we have just ${\bf{H}}({\Theta {\prime} }_{I}|{\Phi }_{I})={\bf{H}}(\Omega )$ where $\Omega$ is the noise.

Thus *all measurement of particle neighbours is eliminated* and Eq. (6) reduces to the two dimensional:

7 $${{\mathcal{T}}}_{gl}^{2D}={\bf{H}}({\Theta {\prime} }_{I}|{\Theta }_{I})-{\bf{H}}(\Omega ).$$

**Figure 1:**
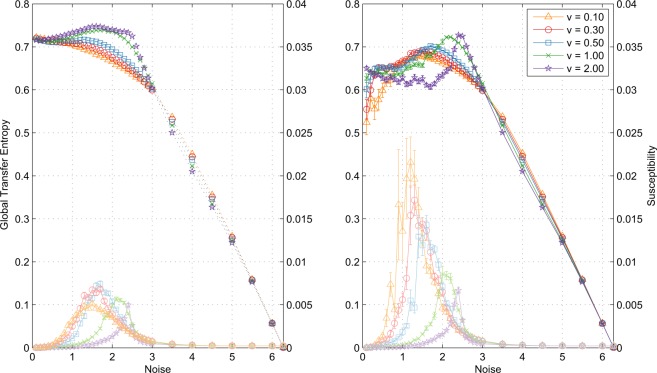
Long-term GTE ${{\mathcal{T}}}_{gl}^{LT}$ (dotted, left) calculated according to Eq. (9) and short-term GTE ${{\mathcal{T}}}_{gl}^{2D}$ (dark lines, right) estimated according to Eq. (7) for a range of particle velocities for $0 < \eta \le 2\pi$. System size $N=1000$ particles, density $\rho =0.25$ and velocities *v* as indicated. Simulation: Long-term ensembles constructed from 20 realisations at observation time $T=500$ time steps each, under ergodic assumptions and the isotropy approximation (see text), while short-term estimated over $T=5000$ time steps (that is, with no ergodic assumption) after relaxation to steady state, using a nearest-neighbour estimator (full simulation details in Supplementary Material). Error bars at 1 s.e. (smaller than symbols) were constructed by 10 repetitions of the experiment (*i.e*., 10 independent ensembles). Lines show susceptibility $\chi$ (Eq. (3)).

Finally, in the long term limit rotational symmetry remains approximately unbroken: that is, for any fixed angle *α*, the joint distribution $({\Theta }_{1}+\alpha ,\ldots ,{\Theta }_{N}+\alpha )$ is the same as the joint distribution $({\Theta }_{1},\ldots ,{\Theta }_{N})$. Under this *isotropy* approximation (see below), Eq. (7) reduces to a one-dimensional form in which only changes in particle heading ${\theta {\prime} }_{i}-{\theta }_{i}$ and noise $\Omega$ appear. Let $p({\theta }_{1},{\theta }_{2})$ be the probability density function (pdf) of $({\Theta {\prime} }_{I},{\Theta }_{I})$ (See Supplementary Material). Under the assumption of rotational symmetry we have:

8 $$p({\theta }_{1},{\theta }_{2})=\frac{1}{2\pi }q({\theta }_{1}-{\theta }_{2}),$$

where $q(\theta )$ is the pdf of ${\Theta }_{I}-{\Theta {\prime} }_{I}$. Since the marginal distributions of ${\Theta }_{I}$ and ${\Theta {\prime} }_{I}$ are uniform on the unit circle in the long-term (ergodic) limit, we obtain ${\bf{H}}({\Theta {\prime} }_{I}|{\Theta }_{I})={\bf{H}}([{\Theta {\prime} }_{I}-{\Theta }_{I}])$ which reduces Eq. (7) to the novel closed-form expression

9 $${{\mathcal{T}}}_{gl}^{LT}={\bf{H}}([{\Theta {\prime} }_{I}-{\Theta }_{I}])-{\bf{H}}(\Omega ),$$

for the long-term GTE, where $[\cdots ]$ denotes the internal angle. Note that at $\eta =2\pi$, ${\bf{H}}([{\Theta {\prime} }_{I}-{\Theta }_{I}])={\bf{H}}(\Omega )=\,\log \,2\pi$ and thus ${{\mathcal{T}}}_{gl}^{LT}$ vanishes at maximum noise, as expected. As noise decreases, the particles align more and more strongly, so that the distributions of both ${\Theta {\prime} }_{I}-{\Theta }_{I}$ and $\Omega$ become increasingly sharply peaked. Since these are both *differential* entropies, they both diverge to −$\infty$. The exact nature of the divergence—as well as the impact of the reduction from Eqs. (7)–(9) (Fig. 1)—is established in simulations discussed below.

The isotropy approximation arises because the SVM on a 2D plane with periodic boundary conditions— *i.e*. a flat torus—is not in fact strictly isotropic. We tested its validity by repeating the long-term simulations while randomly rotating the SVM frame of reference between each update, thus enforcing isotropy^36^. Negligible error was introduced around the phase transition and at very low noise (see Supplementary Fig. S1).

All entropies above are calculated using the continuous estimator developed in^37^ and extended to multiple dimensions in^35,38^ as we previously used for calculating MI in^20^. Conditional entropies of the form ${\bf{H}}(X|Y)$ are calculated using the identity ${\bf{H}}(X|Y)={\bf{H}}(X,Y)-{\bf{H}}(Y)$, and the expanded estimators.

## Results and Discussion

**Figure 2:**
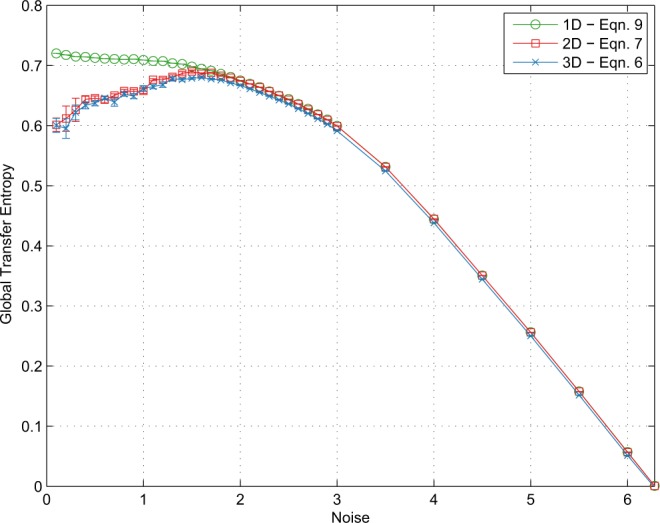
${{\mathcal{T}}}_{gl}$ as measured by the one-dimensional form (Eq. (9), circles), the three-dimensional form (Eq. (6), crosses) and the two-dimensional form (Eq. (7), squares) for $v=0.30$. Error bars at 1 s.e. constructed by 10 repetitions.

Figure 2 (left) shows the long-term GTE ${{\mathcal{T}}}_{gl}^{LT}$ estimated in sample according to Eq. (9) for a range of particle velocities. For $v < 0.5$ there is no peak in the GTE and for $v\ge 0.5$ peaks occur at or below (*i.e.*, low noise regime) the phase transition—identified as a peak in $\chi$ as per Eq. (3) —with all GTE values approaching approximately 0.72 bits as noise tends to zero.

For *short observation times*, by contrast, ${{\mathcal{T}}}_{gl}$ and ${{\mathcal{T}}}_{gl}^{2D}$ estimated according to Eqs. (6) and (7) respectively (and with no isotropy assumption) do peak at the transition, rather than in the high noise regime as in the Ising model^8^; see Figs. 1 and 2 (right).

Figure 1 shows the effect—or lack thereof—of eliminating the consensus vector measurement in Eqs. (6) and (7). The agreement between the two is extremely close, although Eq. (7) gives slightly better results for numerical reasons.

**Figure 3:**
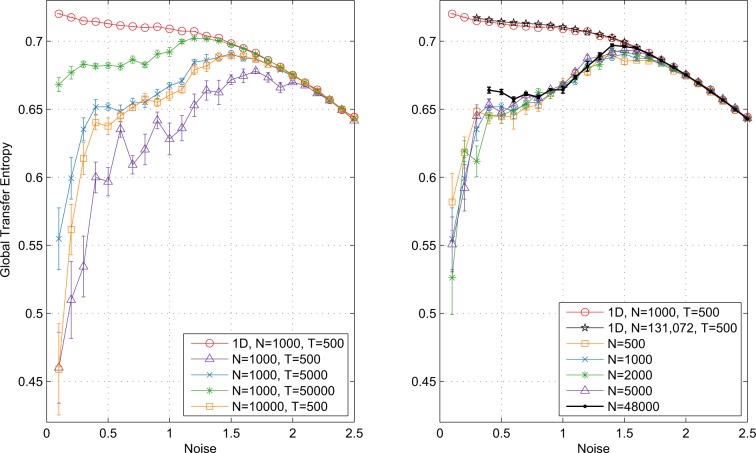
${{\mathcal{T}}}_{gl}^{2D}$ estimates according to Eq. (6) at fixed velocity $v=0.30$ for: left) a range of observation times *T* as indicated and right) varying *N* at $T=5000$ time steps, along with the long-term ${{\mathcal{T}}}_{gl}^{LT}$ of Eq. (9) as per Fig. 2 (left) in both plots. Other simulation details as for previous figures. Left: Note that the blue right triangle line ($N=1000,T=5000$) and orange square line ($N=10000,T=500$) lie on top of each other and represent constant *NT*. Right: Results for large systems (black lines) at low $\eta$ unavailable due to memory limitations in estimation of GTE.

Some flattening at low noise occurs, particularly for higher velocities. Here GTE does not converge to the ~0.72 bits observed in the ${{\mathcal{T}}}_{gl}^{LT}$. The shorter the observation window, the nearer we are to ergodicity-breaking as in the Ising Model^8^ and thus GTE → 0 as $\eta \to 0$. This is confirmed in Fig. 3 (left) which shows ${{\mathcal{T}}}_{gl}^{2D}$ for a single fixed velocity at observation window size varying over two orders of magnitude, along with the long-observation time limit ${{\mathcal{T}}}_{gl}^{LT}$. As observation time increases, the GTE peak flattens and constant GTE in the ordered regime starts to occur, approaching, as predicted, the long-observation time limit.

Finally, Fig. 3 (right) shows the effect of varying the system size. For $\eta  > 0.4$, ${{\mathcal{T}}}_{gl}^{2D}$ increases—converging to ${{\mathcal{T}}}_{gl}^{LT}$—as *N* increases. Below this however, ${{\mathcal{T}}}_{gl}^{2D}$ diverges further as *N* increases, reflecting the reduced capacity of the system to explore large volumes of the phase space. To get a deeper understanding of these results it is useful to consider what is happening in terms of the flock structure. Near the transition, flocks are in flux, breaking apart and reforming. Given this fluidity, a larger number of particles results in more sub-flocks, which consequently are able to explore the phase space more efficiently, so that the GTE approaches ${{\mathcal{T}}}_{gl}^{LT}$. At near-zero noise levels, however, flock stability predominates, with phase space exploration affected mostly by the flock’s random walk-like behaviour  (although it is still possible for flocks to break apart over time)^39^. The magnitude of the random walk is inversely proportional to the number of interacting particles; *i.e*., as the number of interacting particles increases, the mean of the consensus heading at $t+\Delta t$ more closely matches the mean at *t*. Due to the slower random walk, the system explores less of the phase space—more closely approximating ergodicity-breaking—and hence diverges from the long term limit ${{\mathcal{T}}}_{gl}^{LT}$. We also include significantly larger system sizes here, $N=4.8\times {10}^{4},1.3\times {10}^{5}$, demonstrating consistent behaviour with the smaller systems.

Simulation establishes the nature of the aforementioned diverging entropies, showing convergence to ~0.72 bits as $\eta \to 0$ (Fig. 2 (left)), but it is not immediately clear why this value in particular. Analysis of how particle headings evolve over time (see Supplementary Material) reveals an approximate Gaussian distribution of heading differences— *i.e*., Δ$\Theta$—as well as an approximately Gaussian distribution in the heading of the consensus vector—relative to the appropriate particle—as noise tends to zero. From the heading update in Eq. (2) —with particular note of the definition of ${{\mathcal{T}}}_{gl}^{LT}$ in Eq. (9) —we have:

10 $$\begin{array}{rclc}{\theta }_{i}(t+\Delta t) & = & {\varphi }_{i}(t)+{\omega }_{i}(t), & (2\,\mathrm{revisited})\\ {\theta }_{i}(t+\Delta t)-{\theta }_{i}(t) & = & {\varphi }_{i}(t)+{\omega }_{i}(t)-{\theta }_{i}(t), & \end{array}$$

which allows us to decompose Δ$\Theta$ into two independent distributions, defined by ${\varphi }_{i}(t)-{\theta }_{i}(t)$ and ${\omega }_{i}(t)$. The relative consensus heading, ${\varphi }_{i}(t)-{\theta }_{i}(t)$, is approximately Gaussian with support approximately equal to $[-\frac{\eta }{2},\frac{\eta }{2}]$. By definition, noise ${\omega }_{i}(t)$ is uniform with support $[-\frac{\eta }{2},\frac{\eta }{2}]$. By the Central Limit Theorem^40^, summing these two distributions as per the RHS of Eq. (10), yields a truncated Gaussian with range $[\,-\,\eta ,\eta ]$ and variance twice that of the noise; *i.e*., ${\sigma }_{\Delta \Theta }^{2}=2{\sigma }_{\Omega }^{2}$. Empirical results match this, with ${\sigma }_{\Delta \Theta }^{2}=c{\sigma }_{\Omega }^{2}$ where $c\to {2}^{-}$ as $\eta \to 0$ (See Supplementary Table S5).

Thus, closed form entropies for Gaussian and uniform distributions can be substituted into Eq. (9):

11 $$\begin{array}{rcl}{{\mathcal{T}}}_{gl}^{LT} & = & \frac{1}{2}\,{\log }_{2}\,2\pi e{\sigma }_{\Delta \Theta }^{2}-{\log }_{2}\,\sqrt{12{\sigma }_{\varOmega }^{2}},\\  & = & \frac{1}{2}\,{\log }_{2}\,\frac{2\pi ec{\sigma }_{\Omega }^{2}}{12{\sigma }_{\Omega }^{2}},\\  & = & \frac{1}{2}\,{\log }_{2}\,\frac{\pi ec}{6},\end{array}$$

which tends to 0.7546^−^ bits as $c\to {2}^{-}$, in reasonable agreement—given the approximations involved—with the value of 0.72 bits in simulation results given at the beginning of Section 4. shown in Fig. 2 (left) as $\eta \to 0$.

Above the phase transition however, the distribution of ${\Theta {\prime} }_{I}-{\Theta }_{I}$ is no longer approximately Gaussian in nature. As $\eta \to 2\pi$, Δ$\Theta$ becomes increasingly convolved with a uniform distribution before reaching uniformity at $\eta =2\pi$, leading to the steady decrease seen in ${{\mathcal{T}}}_{gl}$ (Fig. 2) over ${\eta }_{c} < \eta  < 2\pi$.

**Figure 4:**
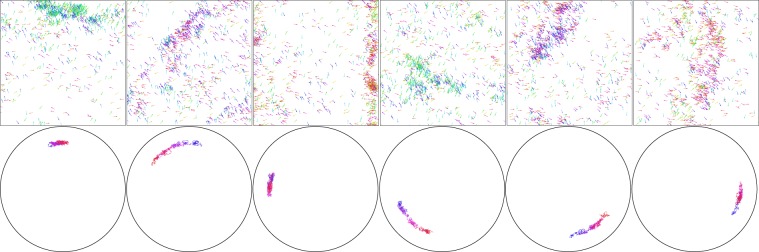
Snapshots from a single simulation demonstrating random walk of heading of high density bands of a flock with $N=1000$ particles at high velocity ($v=2.0$). Snapshots taken at, from left to right, $t=23\times {10}^{3}$, $24\times {10}^{3}$, $28\times {10}^{3}$, $40\times {10}^{3}$, $47\times {10}^{3}$, $49\times {10}^{3}$. Top row shows the state of the flock, while the bottom row shows the two-dimensional order parameter ***M***—that is, mean particle velocity—for the previous 1000 time steps going from blue ($t-1000$) to red (*t*). Distance from the center of the circle corresponds to the order parameter magnitude $M=|{\boldsymbol{M}}|$. Note that, as witnessed by the first two snapshots, the change in heading can be rapid, with only 1000 time steps required for the band to precess *π*/4 radians. Reprinted from^20^.

Observation of the flocks at higher velocities show the appearance of dense travelling bands^27^ as shown in Fig. 4. Finite-size scaling analyses^21^—showing good agreement with theory for susceptibility divergence at the phase transition—also includes this phenomenon. While this could imply symmetry breaking—and therefore no ergodicity, continuously broken or otherwise— Fig. 4 reveals that the high-density band orientation, as well as $\Phi$, performs a random walk through angle space, thus not truly breaking ergodicity. Notwithstanding, the behaviour of the GTE around the phase transition (*i.e*., $1.5\le \eta \le 2.5$) at higher velocities is indeed different to lower velocities (Fig. 2): higher velocities exhibit a peak in both the long- and short-term limits. The appearance of the travelling bands shown in Fig. 4 coincides exactly with this noise/velocity regime, indicating that these bands could be a source of information flow in the flock.

The flat GTE exhibited in the low noise regime is a result of the approximately Gaussian heading of particles relative to their consensus vectors with variance proportional to noise. Although the continuous nature of the SVM cannot be ruled out at this stage, it seems likely that a “discretised” SVM would display similar behaviour with respect to consensus vectors and noise magnitude. In the case of the behaviour of the GTE for the continuous *equilibrium* case, we note that the obvious contender for comparison—the classical XY model—features a *Berezinskii-Kosterlitz-Thouless* (BKT) phase transition (at least in the 2D case)^41^, which would seem to be of an entirely different nature to the transition observed in the 2D SVM model.

Bialek *et al*.^10^ develop a spin-wave approximation for 3-dimensional flocks of starlings, parametrised from real data to explore criticality in flocks. Using this spin-wave model, along with analysis in^9^, Bialek *et al*. discuss long-range order of the velocity (orientation) and speed fluctuations of the flock. At low noise, there is a spontaneous symmetry breaking of the continuous velocity fluctuations, leaving behind a Goldstone mode^42^, wherein there is no energy cost for birds to perform certain changes in flight, which manifests as infinite correlation length^17^. Bialek *et al*. state, however, that there is no spontaneous symmetry breaking in relation to speed fluctuations, therefore no related Goldstone mode; and hence that long-range order of the (speed fluctuations in the) flock must be a consequence of criticality.

Since there are no speed fluctuations in the SVM, we cannot draw any direct conclusions here regarding criticality in real-world flocks. However, our work provides a foundation for further comparative studies of information flow as measured by the GTE in alternative models that *do* feature speed fluctuations, of particular interest is whether of not these other models, such as the spin-wave model of Bialek *et al*. or the Inertial Spin Model (ISM) of Cavagna *et al*.^4^, experience maximal GTE below the transition as seen here.

Information theoretic measures such as GTE can be considered measures of statistical dependency. Specifically, GTE is a measure of the dependence on the previous state of the system, with ${{\mathcal{T}}}_{gl}=0$ iff *X*, conditioned on its own past, is independent of *Y*. The behaviour shown here is that while ${{\mathcal{T}}}_{gl}\to 0$ in the high noise regime—as expected—it in fact remains constant to very low noise: a particle in a low noise flock is just as dependent on its neighbours’ headings as a particle in a flock near the transition. This could be interpreted as another manifestation of continuously-broken ergodicity and the aforementioned Goldstone mode relating to the orientation fluctuations: a particle is still dependent on its neighbours to follow the flock fluctuations about the unit circle, noting that flock fluctuation magnitude is also dependent on observation window size.

Such an interpretation also addresses the differing behaviour to the low temperature Ising model which has no such Goldstone mode. In the equilibrium Ising system below the transition temperature, ergodicity has truly broken with vanishing likelihood of escaping the stable state as temperature decreases, and thus spins become increasingly independent of their neighbours (*i.e*., ${{\mathcal{T}}}_{gl}\to 0$). Furthermore, we have shown that unlike the Ising model, the GTE of the SVM does not peak above the transition temperature.

The Ising model is both a discrete state and equilibrium model whereas the SVM has continuous self-propelled particle velocities and is far-from-equilibrium. Thus it is difficult to determine which of these factors causes the different GTE. Future studies, such as our aforementioned study of the XY model (a continuous system at equilibrium), will address the cause of these differences. The present paper is concerned solely with flocking systems.

While the significant finding in behaviour of the GTE, *maximal information flow from the transition through to very low noise*, has been demonstrated here for the SVM—which is far from the only flocking model, and in fact lacks the speed fluctuations of more realistic models—it seems likely that varying behaviour in the ordered regime will extend to many finite systems which exhibit continuously-broken ergodicity. For these systems, GTE may vary dramatically, although the precise nature of the low noise behaviour is likely dependent on the specific dynamics employed.

## Supplementary information

Supplementary Information.
