# Supplementary material for: Information flow in finite flocks
Source: Sci Rep. 2020 Mar 2;10:3837. doi: 10.1038/s41598-020-59080-6 (PMC7052242; doi:10.1038/s41598-020-59080-6)
Supplement: Supplementary file 1 — Supplementary Information. [file 41598_2020_59080_MOESM1_ESM.pdf]

Supplementary Material for Information flow in finite flocks

J. Brown *[1], T. Bossomaier[2], and L. Barnett[3]

[1]*School of Computing & Mathematics, Charles Sturt University, Bathurst, NSW, Australia*
[2]*Centre for Research in Complex Systems, Charles Sturt University, Bathurst, NSW, Australia*
[3]*Sackler Centre for Consciousness Science, Department of Informatics, University of Sussex, Brighton, U.K.*

15th November 2019

1 The Standard Vicsek Model

The Standard Vicsek Model (SVM) comprises a set of N point particles (labelled $i = 1, \ldots, N$) moving on a plane of linear extent L with periodic boundary conditions. Each particle moves with constant velocity v, and interacts only with neighbouring particles within a fixed radius r, which we take to be 1. Particle positions $\vec{x}_i(t)$ and 2D velocity vectors $\vec{v}_i(t) = (v \cos\theta_i(t), v \sin\theta_i(t))$, where $\theta_i(t)$ is the *heading* of the ith particle[1], are updated at discrete time intervals $\Delta t = 1$. We implement a "backward update" scheme, where both particle positions and velocities for time $t + \Delta t$ are updated on the basis of particle velocities at time t, as opposed to the "forward update" scheme which updates particle positions for time $t + \Delta t$ using the already updated velocity at $t + \Delta t$.

Let $\nu_i(t) \equiv \{j : |\vec{x}_j(t) - \vec{x}_i(t)| < r\}$ be the index set of all particles neighbouring particle i at time t (including i itself, so that $\nu_i(t) \neq \emptyset$). The neighbourhood-average velocity of particle i is then given by:

$$\bar{\vec{v}}_i(t) = \frac{1}{|\nu_i(t)|} \sum_{j \in \nu_i(t)} \vec{v}_j(t) \tag{1}$$

*Email: jbrown@csu.edu.au; Corresponding author

[1]We consider headings as *circular* variables defined on the unit circle $U = (-\pi, \pi]$ with arithmetic modulo 2π.

with heading $\bar{\theta}_i(t)$. Particle positions and headings then update according to Eqn. 1 and Eqn. 2 in the main text respectively, where the $\omega_i(t)$ in Eqn. 1 of the main text are independent and identically distributed (iid) random variables uniform on the interval $[-\eta/2, \eta/2]$, representing (white noise) thermal fluctuations of intensity $\eta \in (0, 2\pi]$. Note that, since a particle travels a distance v in a single time increment $\Delta t = 1$, the SVM only approximates continuity in space and time in case $v \ll 1$ (the model is thus arguably unrealistic as a model for real-world flocking if particle velocities are large). It should be mentioned that while the SVM is defined using discrete time steps, continuous time variants do exist—see [Cavagna et al., 2016] for the numerical integration methods for one such variant. For the sake of pragmatism however, we retain the original discrete approach.

2 The SVM ensemble

We consider the SVM as a *statistical ensemble* of size N, parametrised by the velocity v, particle density[2] $\rho \equiv N/L^2$ and noise intensity η. For simplicity, density is fixed at $\rho = 0.25$ throughout, and noise intensity η is taken as a control parameter. We suppose that the ensemble is relaxed into a steady state, and use capitals $\vec{V}_i, \Theta_i$, *etc.*, to indicate corresponding quantities sampled from the steady-state ensemble. Angle brackets $\langle \cdots \rangle$ denote ensemble averages. In the limit $v \to 0$, the model is equivalent to an XY model, where particles do not move[3], while in the limit $v \to \infty$ particles become fully mixed between updates[Vicsek et al., 1995].

The full order parameter for the SVM ensemble is the 2D random vector

$$\boldsymbol{M} = \frac{1}{Nv} \sum_{i=1}^{N} \vec{v}_i \tag{2}$$

with magnitude $M \equiv |\boldsymbol{M}|$ and heading Φ. We have $0 \leq M \leq 1$, with $M = 1$ if and only if all particles in the ensemble are aligned, and $M \to 0$ in the large-system limit $N \to \infty$. The ensemble variance

$$\chi = \langle M^2 \rangle - \langle M \rangle^2 \tag{3}$$

of the order parameter magnitude defines the susceptibility. Although phase transitions only exists formally in the thermodynamic limit, for finite systems we consider a peak in susceptibility (with respect to a control parameter) as identifying the approximate location of a phase transition.

[2]We consider here the *constant density* ensemble, so that the spatial extent L increases with ensemble size N.

[3]Note that the limiting behaviour of the model as $v \to 0$ must be considered as distinct from models with $v = 0$ [Baglietto and Albano, 2009a].

3 Long-term vs. short-term statistics

In estimating ensemble statistics from simulated (steady-state) dynamics, it is commonplace to invoke *ergodicity* in some form: that is, the simulation is observed, and statistics collated, over a time window of length T, under the assumption that as $T \to \infty$ the statistic in question converges to its ensemble average value. This approach implicitly assumes that observation times are long in comparison to the internal dynamics of the system. In the case of the finite-size SVM, however, this assumption may well be violated, particularly at low noise intensities. What we see, rather, is akin to what has been termed "continuous ergodicity-breaking" [Mauro et al., 2007]: over short observation times, the system is confined to a comparatively small volume of phase space. As we observe the system over increasing lengths of time, progressively larger volumes of phase space are explored. Since a finite SVM is ergodic [Jadbabaie et al., 2003], the system eventually explores the entire phase space. This phenomenon is evidenced by the random walk-like precession of the order parameter heading Φ around the unit circle (*cf.* Fig. 6 in the main article). At low noise, however, observation times necessary to obtain effectively ergodic behaviour become impractically large.

Our resolution to this issue is a pragmatic one: we consider ensemble statistics as essentially observation time-dependent. *Short-term* statistics are thus collated separately (with no ergodic assumptions) over ranges of observation times spanning several orders of magnitude. This affords insights into how the extent of phase space exploration affects our statistics (and also neatly sidesteps the somewhat vexed issue as to whether the SVM features true ergodicity breaking in the thermodynamic limit). In addition, to estimate the limiting ergodic behaviour of the system, we exploit a rotational symmetry approximation to collate *long-term* statistics, under the assumption that in a finite-size SVM symmetry, like ergodicity, is never truly broken.

4 Global transfer entropy from neighbours onto particle headings

Our principal object of study is the global transfer information $\mathcal{T}_{gl}$ from particle's neighbours onto that particle selected uniformly at random from the ensemble, as defined by Eqns. 5-7 and 9 in the main article. In the short-term case, we estimate $\mathcal{T}_{gl}$ over multiple realisations of simulated SVMs. The SVMs are first relaxed/annealed to a steady state, and then headings $\theta_i(t)$ sampled over a further simulation period of T time steps, where T is the observation window. We refer to this as a cooling regime, similar to that which is used in [Vicsek et al., 1995], and further details can be found below in the Simulation section.

We now consider the long-term case. Here we assume *rotational symmetry* of the ensemble; specifically: we assume that for any fixed angle α, the joint distribution of $(\Theta_1 + \alpha, \ldots, \Theta_N + \alpha)$ is the same as the joint distribution of

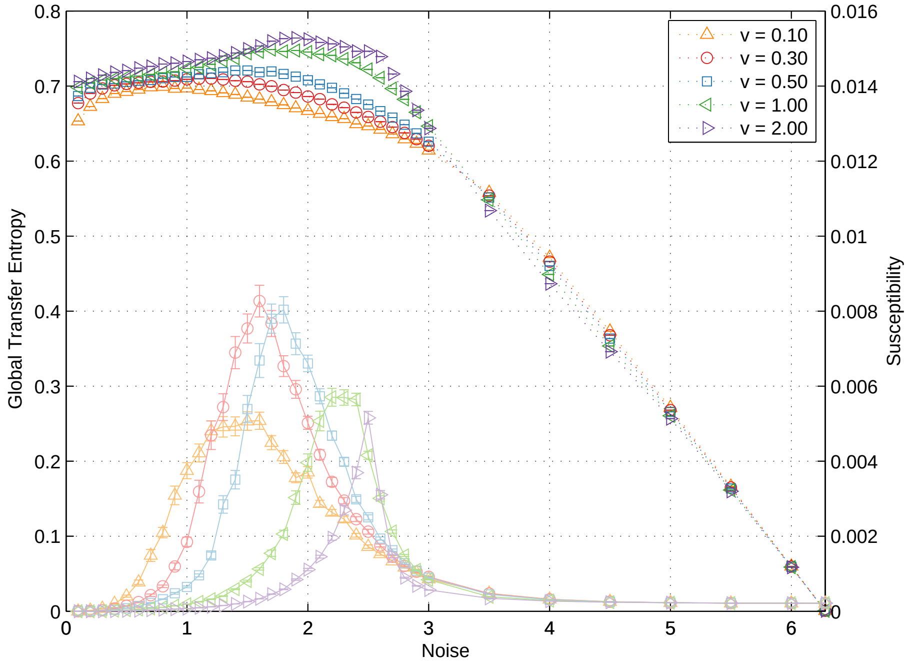

Supplementary Figure S1: Long-term GTE $\mathcal{T}_{gl}^{LT}$ (dotted) calculated according to Eqn. 9 for a range of particle velocities using a rotated reference frame. System size $N = 1000$ particles, density $\rho = 0.25$ and velocities v as indicated. Simulation: 20 realisations at observation time $T = 500$ time steps. Error bars at 1 s.e. (smaller than symbols) were constructed by 10 repetitions of the experiment. Lines show susceptibility χ.

$(\Theta_1, \ldots, \Theta_N)$. We note that the SVM on the 2D torus with periodic boundary conditions is not strictly isotropic, so that this is an approximation. We tested the approximation by repeating our experiments with the frame of reference of the SVM rotated randomly between updates, thus enforcing isotropy [Baglietto and Albano, 2009b]. We found that, in a large, but finite SVM, the isotropy assumption introduces slight error at low noise, likely due to flocks experiencing a shearing effect during the frame rotation procedure—that is, as the frame is rotated and particles adjusted to stay within the system, the number of neighbours and their configurations can arbitrarily change in the space of one timestep. Rotation results can be seen in fig. S1 for comparison with fig. 1 in the main article.

Suppose now that $f(\theta_1, \theta_2)$ is a function of two circular variables with rotational symmetry; that is[4], $f(\theta_1 + \alpha, \theta_2 + \alpha) = f(\theta_1, \theta_2)\ \forall\, \alpha$. Firstly, there exists

[4]Recall that circular variables are defined modulo 2π.

a function $g(\theta)$ such that

$$f(\theta_1, \theta_2) = g(\theta_1 - \theta_2) \tag{4}$$

(this follows from rotational symmetry, setting $\alpha = -\theta_2$) and we may calculate (by a change of variables: $\theta = \theta_1 - \theta_2, \alpha = \theta_1 + \theta_2$) that

$$\iint_{U \times U} f(\theta_1, \theta_2)\, d\theta_1 d\theta_2 = 2\pi \int_U g(\theta)\, d\theta \tag{5}$$

where U denotes the unit circle. Now let $p(\theta_1, \theta_2)$ be the probability density function (pdf) of (Θ_I, Θ_J). Assuming rotational symmetry and setting $f(\theta_1, \theta_2) = p(\theta_1, \theta_2)$ in (4) and (5), we find that

$$p(\theta_1, \theta_2) = \frac{1}{2\pi} q(\theta_1 - \theta_2) \tag{6}$$

where $q(\theta)$ is the pdf of $\Theta_I - \Theta_J$. Now setting $\theta_1 = \theta_I'$ and $\theta_2 = \theta_I$ we obtain for the first term of Eqn. 6

$$\mathbf{H}(\Theta_I' | \Theta_I) = \log 2 + \mathbf{H}(|[\Theta_I' - \Theta_I]|) \tag{7}$$

and the second term is given by

$$\mathbf{H}([\Phi_I + \Omega] \,|\, \Phi_I) = \mathbf{H}([\Phi_I + \Omega], \Phi_I) - \log 2\pi \tag{8}$$

We assume that Ω has zero mean, is symmetric around zero and has support in $[-\pi, \pi]$. Let $\nu(\omega)$ be the pdf of Ω. In our case, Ω is uniform on $[-\frac{1}{2}\eta, \frac{1}{2}\eta]$ with $0 \le \eta \le 2\pi$, so that maximal noise (maximum entropy) is at $\eta = 2\pi$. The pdf is constant, $\nu(\omega) = 1/\eta$, the variance is $\frac{1}{12}\eta^2$ and the entropy is $\log \eta$. Since the noise Ω is *independent* of Φ_I, we have just

$$\mathbf{H}([\Phi_I + \Omega] \,|\, \Phi_I) = \mathbf{H}(\Omega) \tag{9}$$

leading to the expression for $\mathcal{T}_{gl}^{LT}$ given by Eqn. 9 in the main article. Note that $\mathcal{T}_{gl}^{LT}$ vanishes precisely—as expected—when $\Theta_I' - \Theta_I$ is *uniform* on U and thus equal to $\Omega = 2\pi$. At very low noise however, all particles nearly align so that the distribution of $\Theta_I' - \Theta_I$—the differences between each timestep—becomes sharply peaked, just as the distribution of Ω does—see the Average Interactions and Nature of Particle Headings sections below. However, as both $\mathbf{H}(\Theta_I' - \Theta_I)$ and $\mathbf{H}(\Omega)$ are *differential* entropies, these will diverge to $-\infty$ as the variance decreases (*e.g.*, the differential entropy of a narrow uniform "notch" of width ϵ is $\log \epsilon$). We have shown in the long-term observation scenario that these terms diverge *at the same rate*, thus leading $\mathcal{T}_{gl} \to c$ as noise intensity decreases to zero. The convergence is dependent on the nature of $\Theta_I' - \Theta_I$ as discussed in more detail in the Nature of Particle Headings section.

5 Simulation

Simulation models were written in C++ and run on the RAIJIN super cluster at the Australian National Computer Infrastructure Facility. Since the particle velocity (angle) is continuous, the global transfer entropy was calculated using nearest neighbour estimators [Kraskov et al., 2004, Gómez-Herrero et al., 2015]. The accuracy of the estimators was checked by: permutation testing—shuffling the source to remove any information sharing; and decimation—comparing the estimate with subsets of one tenth the number of events [Brown et al., 2017]. Theoretical work on the performance of these estimators is limited and is most relevant to smaller systems [Gao et al., 2016]. The entropy estimation by nearest neighbour is computationally demanding and was carried out in situ on RAIJIN.

To reduce computation times required for simulations to settle into a steady state, we employed a cooling regime, whereby simulations were started with the maximum noise ($\eta = 2\pi$) case, with particles uniformly distributed over the flat torus and headings uniformly distributed on $(0, 2\pi]$. Simulations were run for an initial number T_s of skip steps to allow the system to settle, followed by a data collection phase of T time steps, over which GTE statistics were collated. On completion, η was decreased and another $T_s + T$ simulation steps run with the new η value. This technique enabled reduction of T_s by an order of magnitude, as compared to restarting simulations anew for each η. Appropriate settling time depends on η ($\eta = 2\pi$, for instance, requires zero settling time). We found that a satisfactory regime was to adjust T_s in tiers:

$$T_s = \begin{cases} 1000 & \eta \geq 3.0 \\ 50000 & \eta \leq 1.0 \\ 20000 & \text{otherwise} \end{cases} \tag{10}$$

6 Average interactions

The number of neighbours a particle interacts with at any given time step is clearly influenced by the level of noise—that is, as order and flocking takes hold the number of neighbours within the interaction radius to a particle should naturally increase. Less obvious however are the interaction rates as N and v are varied.

Figure S2 shows that above the phase transition, a difference in N does not impact the average number of interactions. Below the phase transition however, increasing N leads to a sharp increase in how many neighbours a particle interacts with. As we are dealing with metric interactions this measurement is directly correlated with the local density of the flocks. The behaviour in fig. S2 implies that increasing N leads to denser flocks rather than larger—in terms of linear size—flocks. We posit that this behaviour arises from collision mechanics between sub-flocks. That is, when two flocks of roughly equal size collide we see the local density increasing as the two flocks essentially interleave each other. Increasing N thus increases the number of potential flock mergers

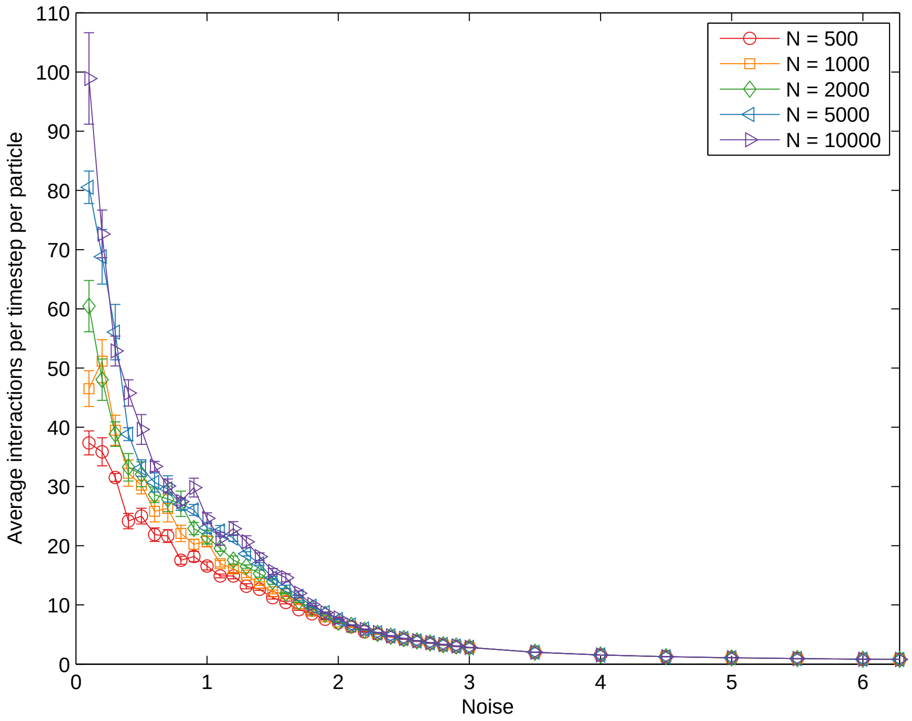

Supplementary Figure S2: Number of average neighbours per particle per time step at varying N at $v = 0.30, T = 5000$.

before a single flock emerges—*i.e.* it would be of order $\log_2 n$ if all sub-flocks were always the same size—thus increasing the highest potential density and thus average interactions.

This is further reinforced when comparing interactions with different velocity values as in fig. S3. In the backwards updating scheme used here, higher velocity particles have difficulty maintaining coherency. More specifically, as v approaches, and overtakes, the interaction diameter $2r = 2$ we see that particles can interact for a single time step before over shooting and immediately leaving each other's interaction zone. Additionally, small perturbations can knock high velocity particles out of alignment and interaction zones. As $\eta \to 0$ however, perturbations are less likely to disrupt flocks enough for density to decrease, so we see a sharp increase in neighbour interactions, approaching that of low velocities.

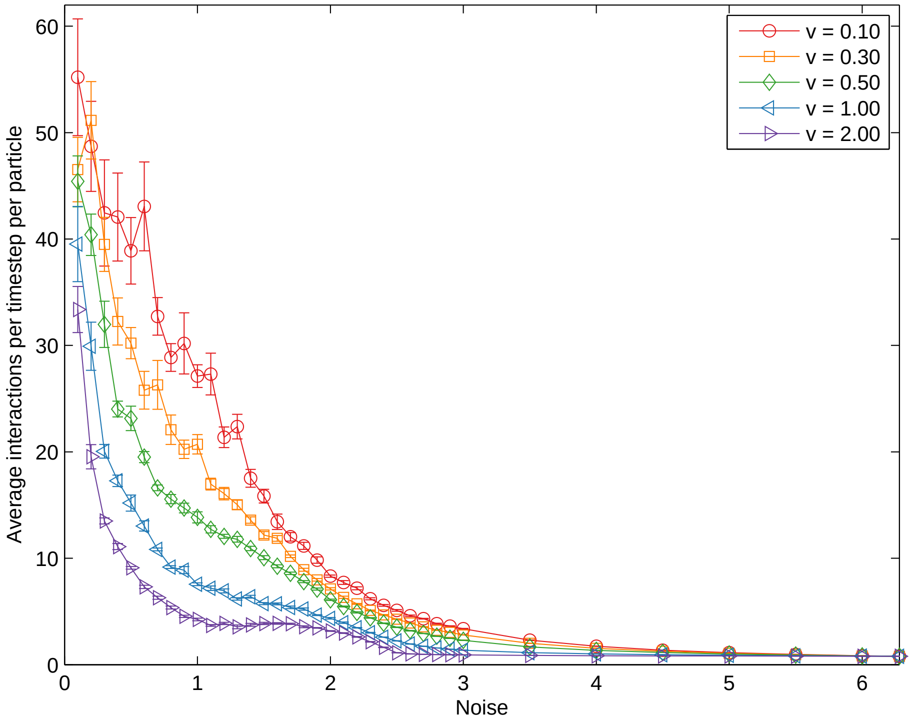

Supplementary Figure S3: Number of average neighbours per particle per time step at varying v with $N = 1000, T = 5000$.

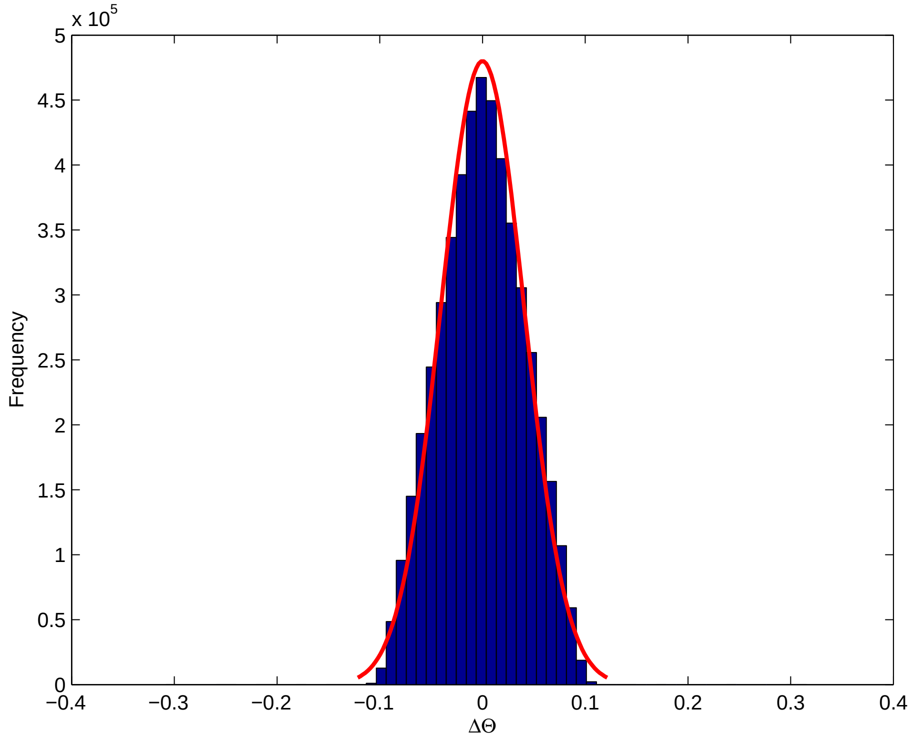

Supplementary Figure S4: Distribution of $\Delta\Theta$ for a single simulation at $\eta = 0.10$ for $N = 1000, T = 5000$ at $v = 0.3$. Line indicates best Gaussian fit. Note the effective a range of values $[-\eta, \eta]$.

7 Nature of particle headings

To understand why $\mathcal{T}_{gl}$ converges to some non-zero value, we analyse the nature of $\Delta\Theta$ as it relates to the noise term, Ω, below the phase transition at η_c. We begin by observing $\Delta\Theta$ in simulation, which reveals a Gaussian distribution with zero mean in fig. S4.

Strictly speaking, since we are dealing with circular statistics, it is in fact a Wrapped Normal distribution, which can be approximated with the von Mises distribution [Mardia and Jupp, 1999]. We include details for the von Mises distribution below, however we note that in the low noise regime, $\Delta\Theta$ does not cross the wrapping boundary at $\pm\pi$ and thus we can continue to treat it as a regular Gaussian variable to keep the problem tractable.

Measurement of the variance of $\Delta\Theta$ and Ω, as shown in Table S5, reveals a convergence of $\sigma^2_{\Delta\Theta} = c\sigma^2_{\Omega}$, where $c \to 2^-$ as $\eta \to 0$. As shown in the main text, substituting the closed form entropy equations into $\mathcal{T}_{gl}$ with these variances leads to a result of 0.7546 bits.

Supplementary Table S5: Variance of $\Delta\Theta$—treating $\Delta\Theta$ as a Gaussian variable ($\sigma^2_{G(\Delta\Theta)}$) and as a von Mises variable ($\sigma^2_{V(\Delta\Theta)}$)—and noise distributions as $\eta \to 0$. Note that $\sigma^2_{\Delta\Theta} = c\sigma^2_{\Omega}$ and that $c \to 2^-$ as $\eta \to 0$, where 2^- denotes that c approaches from the lower side of 2—*i.e.* as η decreases, $c = 2 - \epsilon$.

η	$\sigma^2_{G(\Delta\Theta)}$	$\sigma^2_{V(\Delta\Theta)}$	σ^2_{Ω}
0.1	0.001643	0.0016	0.000833
0.5	0.0405	0.0402	0.2083
1.0	0.160	0.1544	0.0833
2.0	0.603	0.5295	0.33

While this resolves the issue of why $\mathcal{T}_{gl}$ converges to ~0.72 bits, it does not answer why the distribution $\Delta\Theta$ takes the form that it does—that is, Gaussian with twice the variance of the noise. To satisfy this, we must further investigate the components of $\Delta\Theta$.

First, we remember that headings are updated according to:

$$\theta_i(t + \Delta t) = \varphi_i(t) + \omega_i(t) \,, \tag{11}$$

where we recall $\varphi_i(t)$ is the average heading of all neighbouring particles, including i itself. Thus we can transform $\Delta\Theta$ as:

$$\theta_i(t + \Delta t) - \theta_i(t) = \varphi_i(t) + \omega_i(t) - \theta_i(t) \,. \tag{12}$$

By definition, the distribution of $\omega_i(t)$ is uniform with support in $[-\frac{\eta}{2}, \frac{\eta}{2}]$. Less obvious is the nature of $\varphi_i(t)$ and $\theta_i(t)$. In the long term limit—as the SVM performs its random walk—$\theta_i(t)$ becomes uniform, as does $\varphi_i(t)$. We can however sidestep this by considering the distribution of the consensus heading relative to the attached particle—that is, $\varphi_i(t) - \theta_i(t)$. This removes the effect of the random walk and yields the distribution seen in fig. S6 with range $[-\frac{\eta}{2}, \frac{\eta}{2}]$.

This is intuitive, as for a cohesive flock—that is $M = 1$—the consensus vector can only diverge by at most $\pm\frac{\eta}{2}$ radians in one time step, if all connected particles received maximum perturbation in the same direction. Further, reverse our perspective and consider $\theta_i(t)$ relative to $\varphi_i(t)$. Given large neighbourhoods, as demonstrated in the previous section, $\varphi_i(t)$ should remain approximately constant—*i.e.*, $\varphi_i(t + 1) \approx \varphi_i(t)$—and since the new heading of a particle is $\varphi_i(t) + \omega_i(t)$, it stands to reason that the new heading only diverges from $\varphi_i(t)$ by up to $\frac{\eta}{2}$ radians, resulting in the approximate range of $[-\frac{\eta}{2}, \frac{\eta}{2}]$ as shown above.

As per the RHS of equation (12), we now add the distributions of $\varphi_i(t) - \theta_i(t)$ and $\omega_i(t)$. As both have ranges—either exactly or approximately—equal to $[-\frac{\eta}{2}, \frac{\eta}{2}]$ the new distribution by necessity has range $[-\eta, \eta]$, and thus twice the variance of the two component distributions. Finally, as the distribution of $\varphi_i(t) - \theta_i(t)$ is quasi-Gaussian, we expect to see the summed result becoming more Gaussian as per the Central Limit Theorem[5].

[5] Again, we should be accounting for the circular nature of the variables and apply the

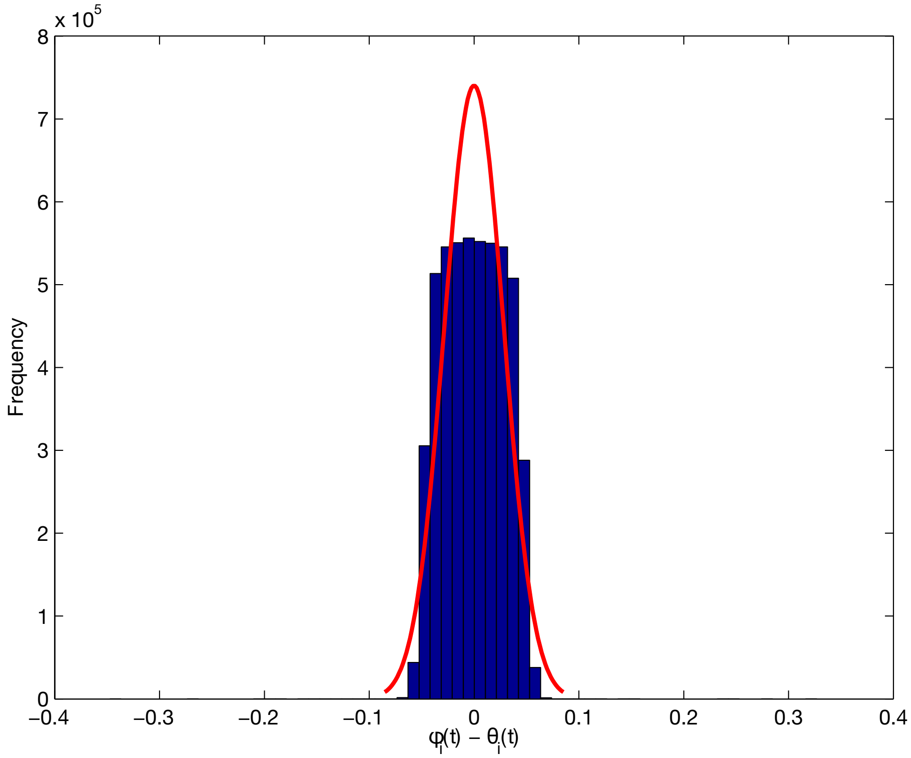

Supplementary Figure S6: Distribution of $\varphi_i(t) - \theta_i(t)$ for all particles in a single simulation at $\eta = 0.10$ for $N = 1000, T = 5000$ at $v = 0.3$. Line indicates best Gaussian fit. Note that while some outliers appear outside of $[-\frac{\eta}{2}, \frac{\eta}{2}]$, ~96.6% of all occurrences lie inside that range.

References

[Baglietto and Albano, 2009a] Baglietto, G. and Albano, E. V. (2009a). Computer simulations of the collective displacement of self-propelled agents. *Computer Physics Communications*, 180(4):527–531.

[Baglietto and Albano, 2009b] Baglietto, G. and Albano, E. V. (2009b). Nature of the order-disorder transition in the Vicsek model for the collective motion of self-propelled particles. *Physical Review E*, 80(5):050103.

[Brown et al., 2017] Brown, J. M., Bossomaier, T., and Barnett, L. (2017). Review of data structures for computationally efficient nearest-neighbour entropy estimators for large systems with periodic boundary conditions. *Journal of Computational Science*, 23(Supplement C):109 – 117.

[Cavagna et al., 2016] Cavagna, A., Conti, D., Giardina, I., Grigera, T. S., Melillo, S., and Viale, M. (2016). Spatio-temporal correlations in models of collective motion ruled by different dynamical laws. *Physical Biology*, 13(6):065001.

[Gao et al., 2016] Gao, W., Oh, S., and Viswanath, P. (2016). Demystifying fixed k-nearest neighbor information estimators. *arXiv prxxeprint arXiv:1604.03006*.

[Gómez-Herrero et al., 2015] Gómez-Herrero, G., Wu, W., Rutanen, K., Soriano, M. C., Pipa, G., and Vicente, R. (2015). Assessing coupling dynamics from an ensemble of time series. *Entropy*, 17(4):1958–1970.

[Jadbabaie et al., 2003] Jadbabaie, A., Lin, J., and Morse, A. S. (2003). Coordination of groups of mobile autonomous agents using nearest neighbor rules. *Automatic Control, IEEE Transactions on*, 48(6):988–1001.

[Jammalamadaka and Sengupta, 2001] Jammalamadaka, S. R. and Sengupta, A. (2001). *Topics in circular statistics*, volume 5. World Scientific.

[Kraskov et al., 2004] Kraskov, A., Stögbauer, H., and Grassberger, P. (2004). Estimating mutual information. *Physical Review E*, 69:066138–066153.

[Mardia and Jupp, 1999] Mardia, K. V. and Jupp, P. E. (1999). *Directional statistics*. John Wiley & Sons.

[Mauro et al., 2007] Mauro, J., Gupta, P., and Loucks, R. (2007). Continuously broken ergodicity. *J. Chem. Phys.*, 126:184511.

[Vicsek et al., 1995] Vicsek, T., Czirók, A., Ben-Jacob, E., Cohen, I., and Shochet, O. (1995). Novel type of phase transition in a system of self-driven particles. *Physical Review Letters*, 75:1226–1229.

directional Central Limit Theorem. However as mentioned earlier, the variables here do not cross the wrapping boundary, and furthermore the directional CLT is simply a convergence to the Wrapped Normal distribution [Jammalamadaka and Sengupta, 2001], thus we continue to treat them as a regular Gaussian variables.
